# Fenugreek seeds, a hepatoprotector forage crop against chronic AlCl_3_ toxicity

**DOI:** 10.1186/1746-6148-9-22

**Published:** 2013-01-31

**Authors:** Yosra Belaïd-Nouira, Hayfa Bakhta, Zohra Haouas, Imen Flehi-Slim, Fadoua Neffati, Mohamed Fadhel Najjar, Hassen Ben Cheikh

**Affiliations:** 1 Laboratory of Histology and Cytogenetic (Research unit of Genetic 02/UR/08-03), Faculty of Medicine, Avenue Ibnou Sina 5000, Monastir, Tunisia; 2 Laboratory of Biochemistry-Toxicology, University Hospital of Monastir, Monastir, Tunisia

**Keywords:** AlCl_**3**_, Liver, Trigonella foenum-graecum, Lipid peroxidation, Histopathology, Iron metabolism

## Abstract

**Background:**

Having considered how bioavailable aluminium (Al) may affect ecological systems and animals living there, especially cattle, and in search for a preventive dietary treatment against Al toxicity, we aimed to test the protective role of fenugreek seeds against chronic liver injury induced by aluminum chloride (AlCl_3_) in Wistar rats.

**Results:**

Five months of AlCl_3_ oral exposure (500 mg/kg bw i.g for one month then 1600 ppm via drinking water) caused liver atrophy, an inhibition of aspartate transaminase (AST), alanine transaminase (ALT) and glutamyl transpeptidase (GGT), an enhancement of both lipid peroxidation and lactate dehydrogenase (LDH) activity and an increase of total protein level in liver. Moreover, histopathological and histochemical examinations revealed moderate alterations in the hepatic parenchyma in addition to a disrupted iron metabolism. Co-administration of fenugreek seed powder (FSP) at 5% in pellet diet during two months succeeded to antagonize the harmful effects of AlCl_3_ by restoring all tested parameters.

**Conclusion:**

This study highlighted the hepatotoxicity of AlCl_3_ through biochemical and histological parameters in one hand and the hepatoprotective role of fenugreek seeds on the other hand. Thus this work could be a pilot study which will encourage farmers to use fenugreek seeds as a detoxifying diet supplement for domestic animals.

## Background

Pollution with the potentially toxic ionic aluminium (Al) is becoming a serious ecological problem related mostly to soil and water acidification caused by acid rain and anthropogenic activities such as overgrazing and overuse of nitrogen and phosphate fertilizers [1]. Indeed, it is well documented that Al dissolves in acid environments which makes it more bioavailable and toxic [2]. Adverse effects caused by high Al levels have been in fact observed in plants and many domestic and wild-life species [3]. Al has also been involved in the etiology of grass tetany, a fatal neuromuscular disease in sheep, goats, and cows [4,5]. Grass tetany is a complex metabolic disorder characterized by hypomagnesemia resulting from mineral imbalances in the diet of ruminants [6]. Post-mortem analyses of rumen contents of cattle who died from grass tetany revealed very high Al contents [7]. It is likely that this disease involves the ability of dietary Al^3+^ to depress serum Mg^2+^ levels in ruminants and its toxic actions in Mg^2+^ metabolism [8]. This has led agricultural scientists to conclude that, if exposure conditions are sufficient, such mineral imbalances in the soil or forage could cause grass tetany. Thus, Al toxicity in cattle was associated with excessive intake of the metal in the diet or contamination of pastures by acidified soils and waters because crops grown on these soils may accumulate high levels of Al [1].

Starting from the fact that chronic exposure to Al is becoming a serious possibility for domestic animals, we aimed to test the ability of fenugreek seeds, a forage crop used as a dietary supplement, to counteract Al toxicity in Wistar rats. In the present study, we focused on the effect of AlCl_3_ on the liver owing to its important role as a detoxifying organ in one hand, and its vulnerability related to its involvement in Al absorption and excretion through biliary flux [9], on the other hand.

Fenugreek (*Trigonella foenum-graecum* L., Fabaceae) is an annual legume crop widely cultivated in Asia, Africa and the Mediterranean countries where it is often used for the edible and medicinal values of its seeds. In other countries like Canada and England, fenugreek is used for incorporation into short-term crop rotation [10] due to its high adaptability to dry climatic conditions, annual nature, and ability to fix atmospheric nitrogen in soil. Thus, fenugreek can enrich soil by fixing N, with benefits to soil conservation and reducing the impact of soil borne pathogens [11]. Fenugreek also provides excellent forage to cattle and can be grown efficiently for hay or silage [12,13]. As a feed for livestock, it is regarded as a bloat free crop that contains growth promoting compounds such as steroids and diosgenin, which are not present in other forage legumes [14]. Current research on fenugreek, most carried for therapeutic purposes, has shown that it contains beneficial chemical constituents including steroidal sapogenins, fiber, galactomannans, antioxidants, and amino acids such as 4-hydroxyisoleucine which possess antidiabetic, antioxidative, hypocholesterolemic, hypoglycemic, anti-inflammatory, antiulcerogenic, antitumor and immunomodulatory properties [15].

The hepatoprotective effect of fenugreek seeds, mainly restricted to studies on ethanol toxicity and diabetes, has also been elucidated through the literature [16-19] but the role of fenugreek in liver against aluminum-induced changes has not so far been considered.

## Results

### Liver weight and total protein level

**Figure 1:**
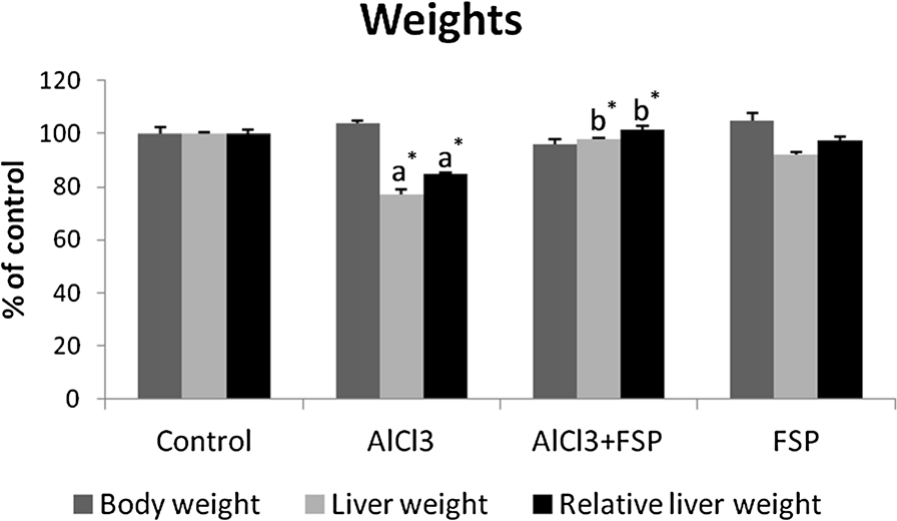
**Effect of FSP on body and liver weights in rats treated with AlCl**_**3**_**.** Values are expressed as means ± SD; n = 10 for each treatment group. ^a^ Significant difference from the control group at p < 0.05. ^b^ Significant difference from the AlCl_3_-intoxicated group at p < 0.05. ^*^ p < 0.001.

**Figure 2:**
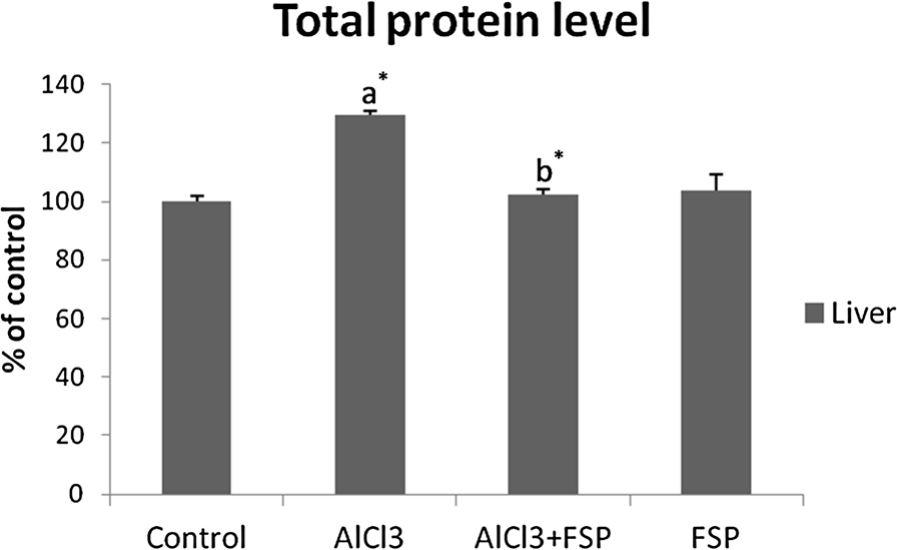
**Effect of FSP on liver total protein level in rats treated with AlCl**_**3**_**.** Values are expressed as means ± SD; n = 10 for each treatment group. ^a^ Significant difference from the control group at p < 0.05. ^b^ Significant difference from the AlCl_3_-intoxicated group at p < 0.05. ^*^ p < 0.001.

No significant difference for the body weight (BW) was observed as compared to the control group. However, a significant decrease in the whole liver weight (22.9%, p <0.001) was observed in AlCl_3_–treated rats. On the other hand, FSP had no effect on the liver weight but succeeded to prevent significantly liver weight loss when administrated with Al (p <0.001). To make a functional comparison of weight gain or loss, tissue weight per 100 g of body weight was calculated. Liver has also shown a significant weight decrease per 100 g of body weight in AlCl_3_–intoxicated group which was reduced to 15% (p <0.001). This relative decrease was lesser after fenugreek administration (p <0.001) (Figure 1). With regard to the total protein level in liver, AlCl_3_ caused a marked increase when compared to the control (+29.69%; p <0.001) and treatment with FSP was found effective to keep a normal value (Figure 2). Treatment with FSP alone had no effect on both liver weight and total protein level.

### Transaminases activities

**Figure 3:**
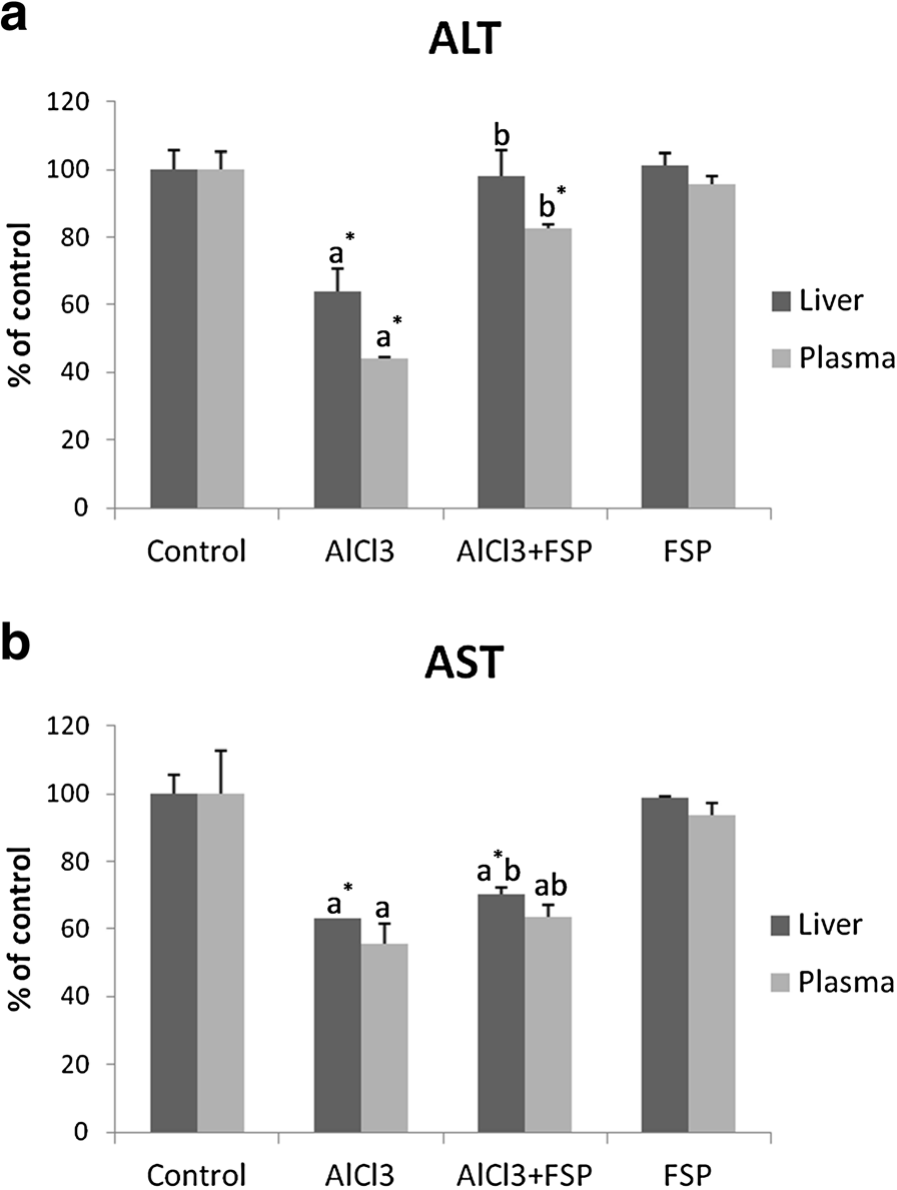
**Effect of FSP on hepatic and plasmatic ALT and AST activities in rats treated with AlCl**_**3**_**.** Values are expressed as means ± SD; n = 10 for each treatment group. ^a^ Significant difference from the control group at p < 0.05. ^b^ Significant difference from the AlCl_3_-intoxicated group at p < 0.05. ^*^ p < 0.001.

The activities of ALT and AST in both liver and plasma were significantly decreased in rats treated with AlCl_3_ (−36%, -36.89% and −55.7%, -44.29% respectively). The administration of FSP with AlCl_3_ restored the normal level of plasmatic and hepatic ALT activity and succeeded to increase AST activity by +7.1% in liver and +7.6% in plasma (Figure 3 (a) and Figure 3 (b)).

### GGT activity as a cholestasis marker

**Figure 4:**
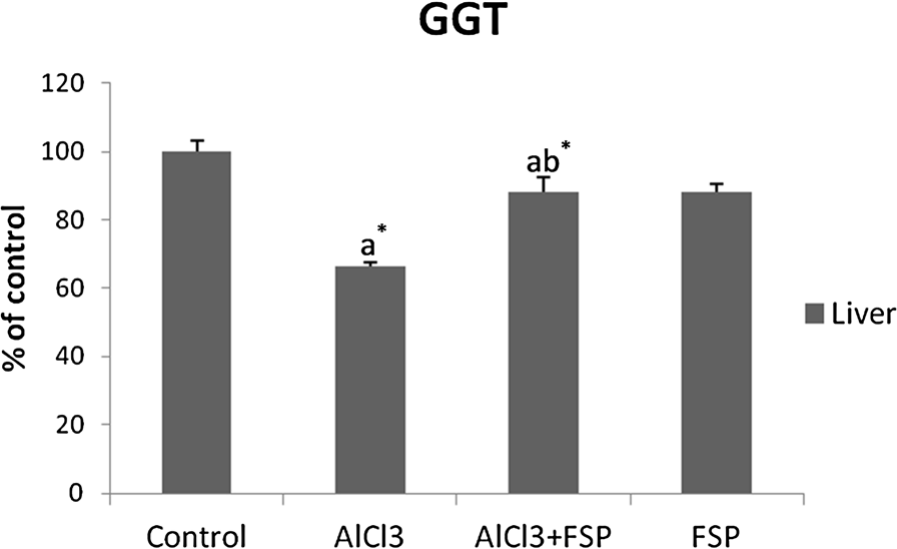
**Effect of FSP on hepatic GGT activity in rats treated with AlCl**_**3**_**.** Values are expressed as means ± SD; n = 10 for each treatment group. ^a^ Significant difference from the control group at p < 0.05. ^b^ Significant difference from the AlCl_3_-intoxicated group at p < 0.05. ^*^ p < 0.001.

As it is shown in Figure 4, AlCl_3_ decreased GGT activity by −33.67% (p <0.001) but FSP supplementation ameliorated it registering an increase of +21.9% (p <0.001).

### Plasma markers

Data presented in Table 1 showed that treatment with AlCl_3_ caused a significant increase in blood glucose (+97.8%; p <0.001). Given with AlCl_3_, FSP decreased plasma glucose to the normal value. On the other hand, a significant hypercholesterolemia together with a significant hypertriglyceridemia (p <0.001) were noticed during AlCl_3_ intoxication but fenugreek seeds were able to re-establish the normal values.

**Table 1:** **Effects of FSP on some plasma markers (Glucose, cholesterol and triglycerides) in rats treated with AlCl**_**3**_

**Parameter**	**Experimental groups**			
	**Control**	**AlCl**_**3**_	**AlCl**_**3**_**+FSP**	**FSP**
Glucose (mmol/l)	8.39 ±0.03	16.6^a*^±1.30	8.18^b*^±0.08	8.31 ±0.02
Cholesterol (mmol/l)	1.56±0.009	2.22^a*^±0.36	1.56^b*^±0.01	1.56±0.08
Triglycerides (mmol/l)	0.66±0.02	1.20^a*^±0.06	0.58^b*^±0.02	0.58±0.14

Values are expressed as means ± SD; n = 10 for each treatment group.

^a^ Significant difference from the control group at p < 0.05.

^b^ Significant difference from the AlCl3-intoxicated group at p < 0.05.

^*^ p < 0.001.

### Lipid peroxidation estimation in liver tissue

**Figure 5:**
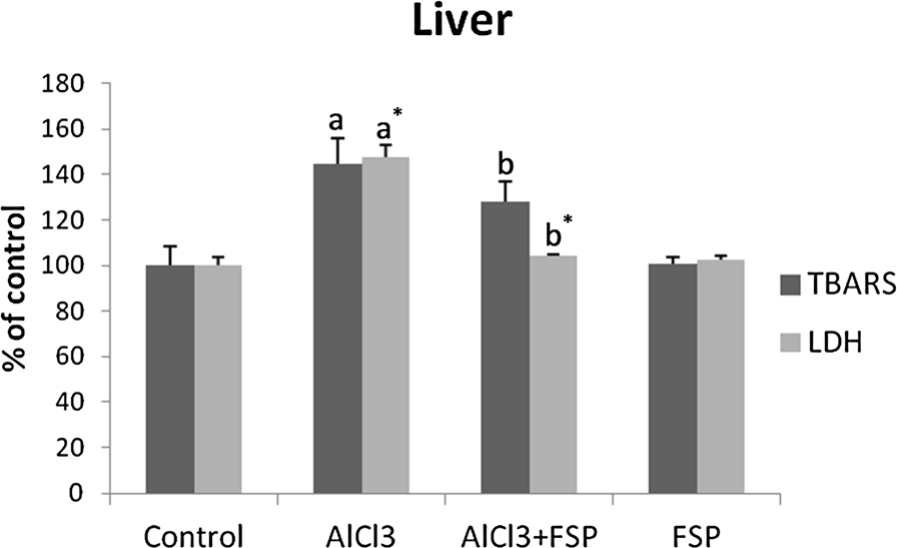
**Effect of FSP on hepatic TBARS and LDH levels in rats treated with AlCl**_**3**_**.** Values are expressed as means ± SD; n = 10 for each treatment group. ^a^ Significant difference from the control group at p < 0.05. ^b^ Significant difference from the AlCl_3_-intoxicated group at p < 0.05. ^*^ p < 0.001.

After AlCl_3_ administration, TBARS (Thiobarbituric Acid Reactive Substances) level was increased by +44.6% compared to the controls and consecutively LDH activity raised by +47.4%. However, when FSP was given with AlCl_3,_ we noticed a decrease of TBARS level by −16.7% and LDH activity by −42.9% (Figure 5).

### Histopathological and histochemical results

**Figure 6:**
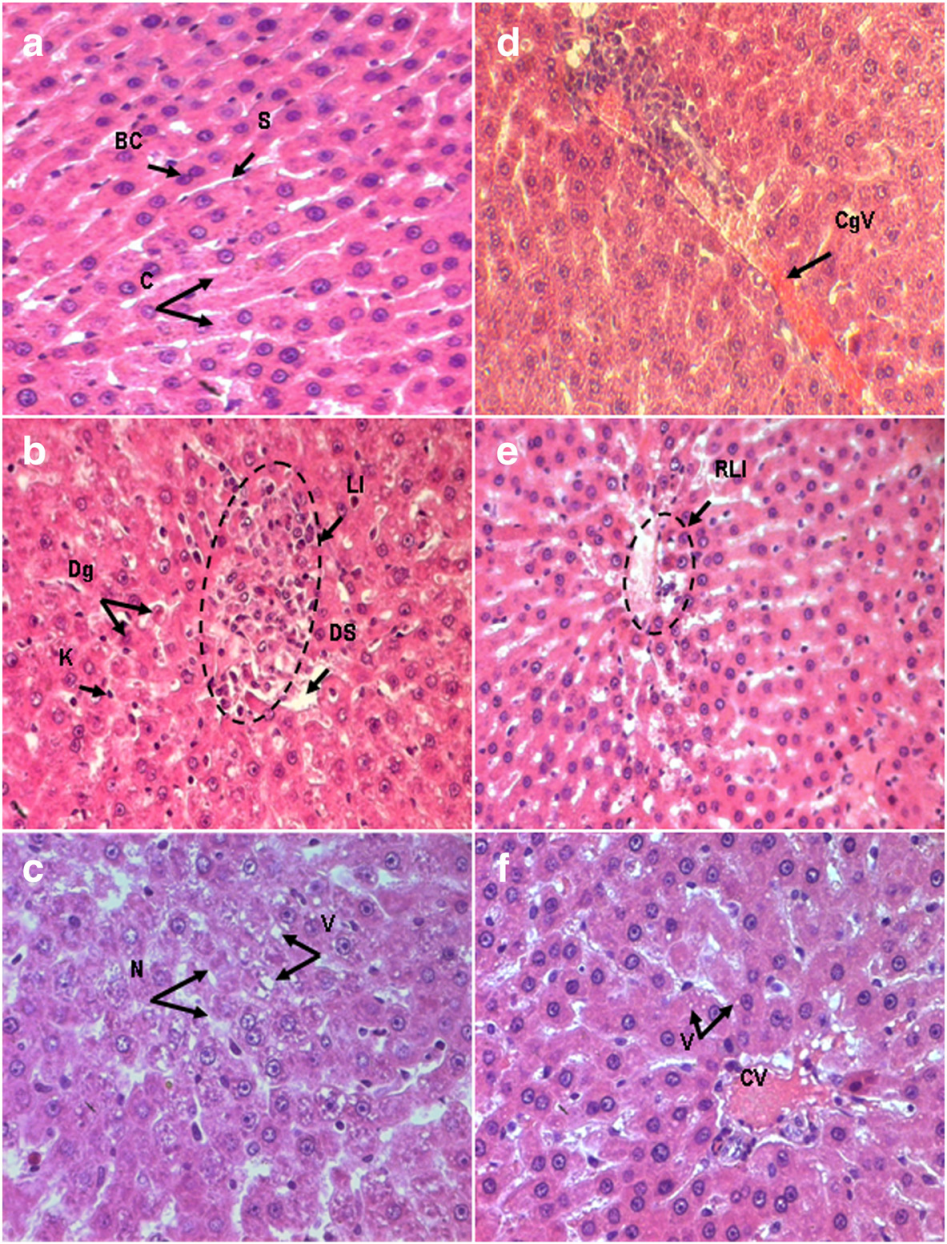
**Light micrographs of rat liver tissue stained by hematoxylin-eosin (HE) in control (a)(x500), AlCl**_**3**_**-treated group (b,c,d)(x320, x500, x500 respectively) and Fenugreek+AlCl**_**3**_**-treated group (e, f) (x320, x500 respectively).** C: hepatic cords, BC: binucleated cell, S: sinusoids, Dg: degenerating cell, K: Kupffer cell, DS: dilated sinusoid, LI: lymphoid infiltrate, N: necrotic cells, V: vacuoles, CgV: congested vein, CV: central vein.

Liver tissue from control rats (Figure 6 (a)) depicts a typical normal liver histology and hepatocyte structure with normal lobular architect and hepatocytes arranged in cords encircling the central canal. Similarly, sections of FSP-treated groups showed normal arrangement of hepatocytes and sinusoids. In rats administered AlCl_3_ (Figures 6 (b), 6 (c) and 6 (d)), the hepatic injury was marked by moderate hepatocellular necrosis randomly distributed throughout the parenchyma, as well as an increase in inflammatory cell infiltration, vascular congestion, pyknotic nuclei, moderate cytoplasmic vacuolation, granulous aspect of cytoplasm and dilated sinusoids. Changes were improved in FSP post-treated rats (Figures 6 (e) and 6 (f)), which exhibited areas of normal liver architecture, reduced cytoplasmic vacuolation and centrilobular necrosis in addition to normal sinusoidal spaces and less granulous aspect of cytoplasm. Groups treated with FSP after exposure to AlCl_3_ were also characterized by an increased number of bi-nucleated cells.

**Figure 7:**
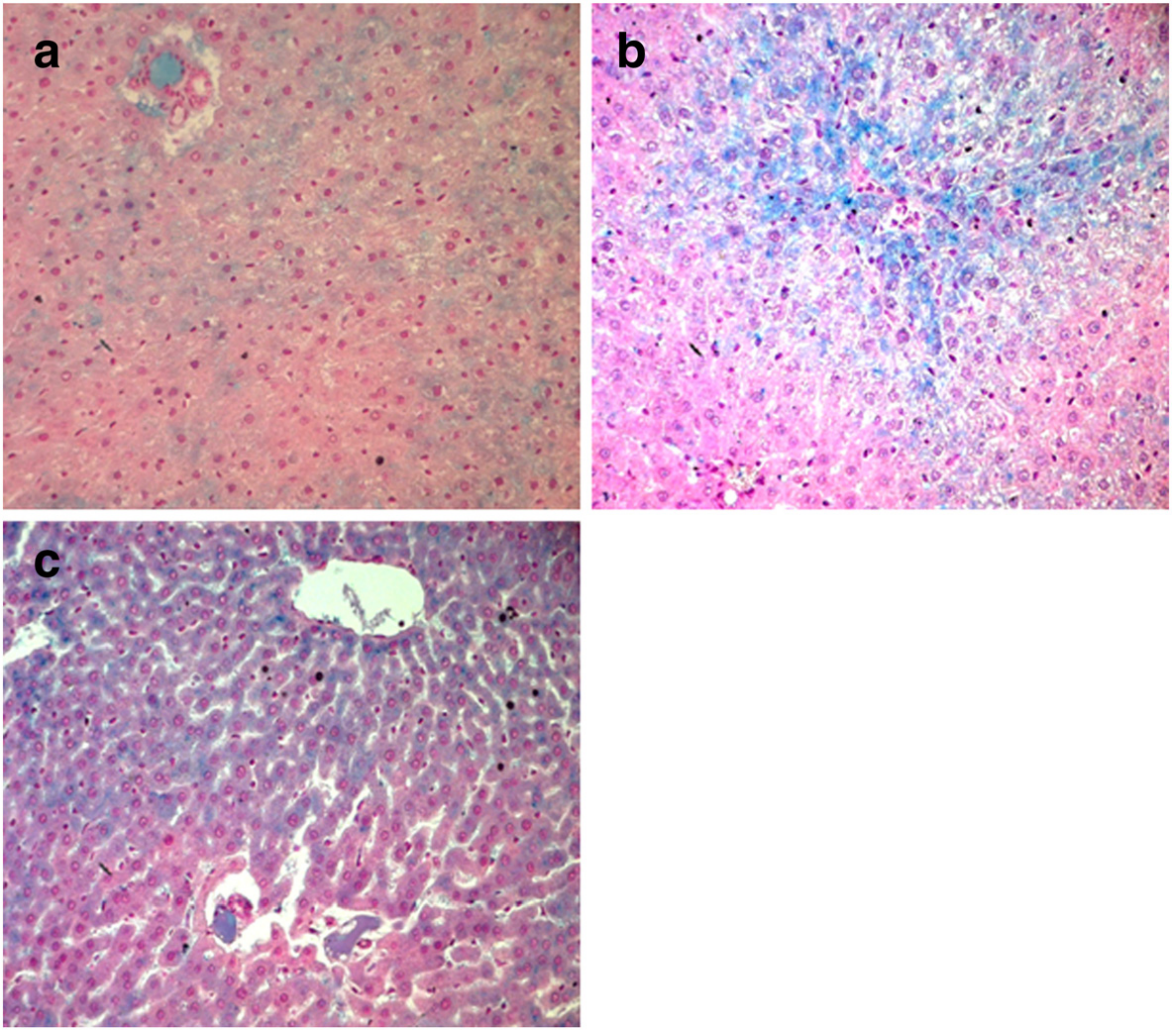
**Light micrographs of Perl’s staining of rat liver tissue in control (a), AlCl**_**3**_**-treated group (b) and Fenugreek+AlCl**_**3**_**-treated group (c) (x100).**

Iron distribution in paraffin sections of rat liver stained with Perl’s is illustrated in Figure 7. Section of control hepatic parenchyma (Figure 7 (a)) showed a slight deposition of iron in liver tissue homogeneously distributed. Hepatocytes localized around the central vein and around portal areas accumulated a larger quantity of iron. After Al intake, an abnormal and more pronounced Perl’s positive iron deposition was detected in pericentral and parenchymal cells compared to the control group (Figure 7 (b)). Treatment with FSP restored the normal aspect of iron distribution (Figure 7 (c)).

## Discussion

In Al-treated animals, the hepatic weight loss is an indicator of Al toxicity as it was mentioned in another report [20]. It may be due to enhanced catabolic processes such as glycogenolysis, lipolysis or proteolysis, which is the outcome of lack of cellular glucose in liver cells. The disturbance of the catabolic process in the liver was evidenced in this study by a significant hyperglycemia and an altered lipid metabolism (hypercholesterolemia and hypertriglyceridemia). Besides, liver atrophy could be understood if we consider the similarities of Al hepatotoxicity to the aging process evoked by Stacchiotti et al. (2008) [21]. In fact, a positive relationship between smaller organ mass and senescence has been proven [22].

On the other hand, a remarkable increase of hepatic total protein level was noticed. This is because Al, like other toxic metals, may affect intracellular processing of secreted proteins and also retard their discharge, resulting in the inhibition of protein secretion from liver parenchymal cells [23]. This result may explain the decrease of plasma total proteins in rats intoxicated by AlCl_3_ recorded in other reports [24,25]. Moreover, based on the correlation established by Stacchiotti et al. (2008) between Al toxicity and liver aging [21], this result could also be explained by the increase in total protein synthesis, typical to advanced age, which may be due to a compensation by the liver for a more pronounced proteinuria, increased proteolysis or an accumulation of “altered” proteins [26]. This was evidenced in this study by the elevation of intra-hepatic LDH. Within this context, it was reported that Al was able to enhance the synthesis of some stress proteins (HSP 72 and GRP 75) in liver after chronic exposure [27].

The concomitant treatment of rats by FSP succeeded to prevent liver weight loss and was able to reduce total protein level probably due to fenugreek seeds capacity to correct both glucose and lipid metabolism and prevent lipid peroxidation as it was shown in this study and may be modulating the repair protein battery. The role of a possible regenerative process could also be considered [28].

Regarding histopathological changes induced by AlCl_3_ in liver, it is assumed that Al exposure can result in Al accumulation in liver cells [29], where it produces dose-dependent cytotoxic effects [30]. Our results demonstrated that AlCl_3_ caused structural changes in the liver tissue of Wistar rats recalling those generated by other hepatotoxins [31]. Al treatment generated cytoplasmic vacuolation evoking the hepatosteatosis, known by an excess of lipids accumulation in cytoplasmic vesicles. In fact, it was found that Al toxicity enhances lipid accretion in hepatocytes [32] due to the impairment of lipid metabolism as it was shown by the increase of both total cholesterol and triglycerides and particularly the increase of lipid peroxidation as it was evidenced by increased hepatic TBARS (Thiobarbituric Acid Reactive Species) level. Besides, the granular aspect of the cytoplasm after AlCl_3_ treatment could be the consequence of hemosiderin deposit, as attested by Perl’s staining performed in this study and other reports [21]. Hemosiderin accumulation is in fact a result of iron metabolism impairment as a consequence of AlCl_3_ toxicity [33].

Against all these pathological changes induced by AlCl_3_ in liver tissue, FSP administration was found effective. Indeed, we noticed a reduction in the size of inflammatory cell infiltration areas attributable to the proven anti-inflammatory activity of *Trigonella*[34] and a reduction in the number of necrotic cells. Interestingly, it has been shown that methanol fenugreek seed extract was able to decrease 3-NT (3-nitrotyrosine) immunostaining, a marker of protein damage and a biomarker of inflammation, following ethanol toxicity *in vivo*[16]. The ability of fenugreek to prevent necrosis was already proven in primary rat hepatocytes culture against N-methyl-N_-nitro-N-nitrosoguanidine (MNNG) toxicity [35]. A reduction of lipid vacuoles was also observed in this study, which reflects an improvement of lipid metabolic disorders generated by AlCl_3_ especially the decrease of lipid peroxidation. Interestingly, we found that liver section of Al-intoxicated rats treated by fenugreek exhibited increased mitosis giving evidence of a regenerative process that might be related to an effect on interleukin-6 [28].

In parallel with histopathological evaluation, liver function tests were carried out. Al has been known to increase plasmatic levels of AST and ALT in association with cellular degeneration of the liver [24,25]. Unexpectedly, we noticed a decrease of ALT and AST levels in liver homogenates that did not reflect its escape to blood because their plasmatic levels decreased too. Moreover, histological alterations were not enough drastic to cause a complete depletion of these enzymes. Consequently, it seems that 5 months of AlCl_3_ treatment have inhibited ALT and AST activities. Transaminases inhibition has been described in other reports as a mechanism of toxicity of some chemicals such as fenthion [36], ethanol [37], hydrazine [38] or acetaldehyde [39]. This result, although unexpected, supports the postulate that inhibition of transamination might play a role in the chronic AlCl_3_ toxicity on protein and carbohydrate metabolisms as previously demonstrated [24]. In fact, it was published that Al ions could inhibit pyridoxal phosphate catalyzed decarboxylation and transamination *in vitro*[40] and it is worthwhile to mention that ALT and AST are pyridoxal phosphate (PLP)-dependent transaminase enzymes.

As a marker of cholestasis, liver GGT was assayed. Specific to biliary ducts, these enzymes are expected to decrease pathologically in liver tissue. In the present study, a significant decrease was observed in intra-hepatic GGT activity, which means that 5 months of AlCl_3_ intoxication could cause cholestasis.

With regard to the protective role of fenugreek seeds against AlCl_3_-induced changes in biochemical parameters, it is upheld that FSP can ameliorate hepatic function through the significant amelioration of ALT and AST activities in the liver and plasma as well as the prevention of cholestasis.

Then again, the increased levels of TBARS suggest that AlCl_3_ exerts a significant oxidative stress through lipid peroxidation on liver tissue. This result is perfectly concordant with other reports which elucidated lipid peroxidation as a major mechanism of Al salts toxicity in the liver and other organ cells [25,41]. Increased lipid peroxidation could be a result of various mechanisms such as affecting the activity of antioxidant enzymes [42], inducing glucotoxicity through pancreatic beta-cell dysfunction and insulin resistance [43] or disturbing cellular metal homeostasis especially that of iron [44]. Knowing that iron is stored mostly in the liver as ferritin or hemosiderin, Perl’s staining was performed. In this study, Al reduced liver iron stores and caused an abnormal and diffused iron hemosiderin Prussian blue deposit within pericentral lobular hepatocytes. This could be related to altered cellular homeostasis of iron already proven [45]. Indeed, Al ions could replace iron and magnesium ions in the biological systems leading to Al overloading in different organs. Free iron ions are able to initiate cellular damage [46]. Al could also enhance iron uptake into ferritin, the major iron storage protein localized in the liver together with transferrin receptors, and its conversion to hemosiderin [45]. Our histochemical results are concordant with earlier published data [21,27].

FSP was able to protect liver from lipid peroxidation induced by AlCl_3_. It is likely that lipid peroxidation in the liver is owing to the antiradical and antioxidant potential of *Trigonella* seeds emphasized through *in vitro* and *in vivo* experiments [16,47-50]. Fenugreek seeds are in fact rich in polyphenolic flavonoids (>100 mg/g) [51] and recently quercetin, one of the identified flavonoids in fenugreek seeds, was found able to protect rat hepatocytes against oxidative damage induced by ethanol [52]. The well known hypoglycemic property of fenugreek seeds could also be behind its anti-peroxidative action in liver as it was proven during diabetes [53]. The effect of fenugreek seeds on iron metabolism might also be proposed as a hepatoprotector mechanism since FSP restored the normal iron distribution feature in liver. This result corroborates the findings of other authors who noticed the ability of the seed extract to prevent iron-induced lipid peroxidation *in vitro*[54].

## Conclusions

Even if preliminary, this study has illustrated the curative role of fenugreek seeds against Al-induced hepatotoxicity. This work is an additional result in favor of this multi-purpose plant. It could be a motivating work for farmers and industrials to incorporate at reasonable rates fenugreek seeds, cheap and easy to obtain, in livestock feed in order to protect cattle from chronic aluminium accumulation.

## Methods

### Reagents

Aluminum chloride ((AlCl_3_, 6H_2_O), analytical grade) was purchased from Sigma-Aldrich Chemical Co. (St. Louis,USA). All other chemicals were of analytical grade.

### Animals

Wistar rats (weighing 208–220 g) were obtained from the Central Pharmacy (SIPHAT, Tunis, Tunisia). They were fed pellet diet purchased from the Industrial Society of Rodents’ Diet (SICO, Sfax, Tunisia) and tap water *ad libitum*. Animals were kept in an air-conditioned room (temperature 22 ± 3°C and relative humidity of 40%) with a 12h light/dark cycle. The experimental procedures were carried out according to the National Institute of Health Guidelines for Animal Care and approved by the local Ethics Committee (The Tunisian Association of Laboratory Animals Sciences (ATSAL, Visa 2007T02602APSF1 J.O.R.T. 27 April 2007 n°34. p 2115).

### Preparation of fenugreek seed powder (FSP)

*Trigonella* seeds purchased from the local market were finely powdered and mixed at 5% in ground standard rat feed (i.e. 5 g of dry ground *Trigonella* seeds in 95 g of ground rat food). The dose of fenugreek seeds employed in this study was chosen according to previous studies and has been subjected to nutritional and safety evaluation [55].

### Study design

Rats were treated according to the modified protocol established by Gong et al. [56]. In brief, rats were randomly distributed into four groups of ten animals each: control; AlCl_3_ daily during 5 months at the level of 500 mg/kg bw i.g for one month then 1600 ppm via drinking water; AlCl_3_ plus fenugreek seed powder at 5% in standard rat food (FSP) during the last 2 months and FSP alone.

### Blood and tissue collection

Blood samples were collected under anesthesia by cardiac puncture in heparinized tubes. Plasma was obtained by centrifuging the blood at 3,000 *xg* for 15 min at 4°C and stored in aliquots at −20°C until analysis. Livers were removed quickly from animals, washed in ice-cold physiological saline and weighed. Then, multiple lobes of the liver from each rat were cut out. Some of them were fixed in Bouin’s fluid and embedded in paraffin; others were minced and homogenized (10% w/v) separately in ice-cold 1.15% KCl-0.01mol/L sodium, potassium phosphate buffer (pH 7.4) in a Potter-Elvehjem type homogenizer. The homogenate was centrifuged at 10,000*xg* for 20 min at 4°C, and the resultant supernatant was stored at −80°C to be used for different assays.

### Histological studies

Cross sections of paraffin-embedded livers were cut (5 μm thicknesses) and stained with either hematoxylin and eosin (HE) for light microscopy examination [57] or Perl’s reagent to reveal iron distribution [58].

### Biochemical assays

#### Assays of liver and plasma markers

Total proteins, ALT, AST, GGT, total cholesterol, triglycerides, LDH and glycemia, were determined using enzymatic methods on Integra 400 plus™ (Roche Diagnostics, products references are #03183734190, #20764957322, #20764949322, #03002721122, #03039773190, #20767107322, #03004732122 and #04404483190 respectively).

#### Lipid peroxidation estimation

The extent of lipid peroxidation in liver was assessed by measuring the content of thiobarbituric acid reactive substances (TBARS) following the method of Buege and Aust [59]. TBARS were expressed as malondialdehyde (MDA) amount using freshly diluted malondialdehyde bisdimethylacetal as standard.

### Statistical analysis

Data were expressed as mean ± standard deviation (SD) and analyzed using one-way analysis of variance (ANOVA) followed by the post hoc Tukey’s test, used for comparison. Values were considered statistically significant when p < 0.05. Statistics were done using IBM SPSS Statistics 19.

## Competing interests

The authors declare that they have no competing interests.

## Author’s contributions

YB-N conceived this study, designed it, analyzed and interpreted the data and wrote the manuscript. HB participated in the study design and data acquisition. ZH took the pictures of histological sections and interpreted them. FN has made substantial contributions in biochemical assays. IF-S carried out some of the plasmatic assays. MFN and HBC drafted and revised the manuscript. All authors read and approved the final manuscript.
